# Reliability and validity of the repetitive behavior scale-revised for young Chinese children with autism spectrum disorder in Jiangxi Province

**DOI:** 10.3389/fped.2022.939841

**Published:** 2022-09-08

**Authors:** Xiu Luo, Yaoyao Xiong, Mei Gu, Liyun Huang, Zhonghui Lu, Xia Zhong, Shipu Zou

**Affiliations:** Department of Children Health, Jiangxi Provincial Children’s Hospital, Nanchang, China

**Keywords:** restricted and repetitive behaviors (RRBs), Repetitive Behavior Scale-Revised, autism spectrum disorder (ASD), confirmatory factor analysis (CFA), validity, reliability

## Abstract

Restricted and repetitive behaviors (RRBs) are one of the two main diagnostic features of autism spectrum disorder (ASD). To date, a growing body of research on RRB in children with ASD has recently attracted academic attention. The Repetitive Behavior Scale-Revised (RBS-R) was primarily intended for use in evaluating RRBs observed in ASD. This study recruited 381 Chinese children with ASD aged 2–4 years to measure the reliability and validity of the RBS-R. Confirmatory factor analysis (CFA) was applied to the structuring models of the four proposed structural models, indicating that a 6-factor model demonstrated good internal consistency and the best fit based on common overall fit indices. These findings suggest the utility of the Chinese version of RBS-R.

## Introduction

Autism spectrum disorder (ASD), defined as a neurodevelopmental disease, has two core symptoms including social communication deficits and restricted and repetitive behaviors (RRBs) and sensor stimuli behaviors (1). Genes, environment, and the interaction between genes and environment are the etiology causes of ASD (2). The Autism and Developmental Disabilities Monitoring (ADDM) network estimated the prevalence of ASD among children aged 8 years, showing 0.66% in 2002 (3), 1.46% (4) in 2012, 1.68% (5) in 2014, and 1.85% (6) in 2016, respectively. A Chinese nationwide multi-center population-based study (7) recruited 125,806 participants aged 6–12 years to determine the prevalence of ASD, revealing that 0.7% of these children were diagnosed with ASD. This figure was higher than the 0.118% reported in a meta-analysis conducted in China (8), which included 25 studies correlated with the prevalence of ASD. As the prevalence of autism increases, there is an increasing focus on the condition around the world.

Restricted and repetitive behavior is a series of behaviors characterized by high frequency and invariant repetition; this results in the child desiring environmental sameness (9). RRBs have four subtypes of behavior in the Diagnostic and Statistical Manual of Mental Disorders 5th edition (DSM-5). The first is the repetition of actions, speech, or use of objects, such as turning around and parroting. The second is insisting on sameness such as walking the same route. The third behavior is restricted interests, including obsessing with wheels. The last one is sensory processing abnormalities, for example, smelling people’s hair (10). It is well known that the social communication of ASD has gained considerable research attention over the years; however, the field of RRBs lags far behind the former. In many infants later diagnosed with ASD, RRBs are detectable after 12 months of age, which may be some of the earliest detectable behavioral markers of ASD (11). RRBs are also present in typically developing (TD) infants, toddlers, and developmental delay (DD) children (12). Through literature review, we found that most of the measurements of RRBs were derived from some items of the Autism Diagnostic Interview-Revised (ADI-R) (13) and the Autism Diagnostic Observation Schedule (ADOS) (14) and a scale named the Repetitive Behavior Scale (Revised Edition), a 43-item informant-based rating scale, was used to assess RRBs only. This scale includes six subscales: (a) Rituals, (b) Self-injurious Behavior, (c) Stereotypic Behavior, (d) Compulsive Behavior, (e) Restricted Interests, and (d) Sameness. The RBS-R aimed to measure the presence and severity of a variety of RRBs and its items were drawn from other instruments and conceptually categorized based on the authors’ clinical experience (15). Numerous investigations have been conducted on the psychometric properties of the RBS-R including in Canada (16), German (17), Italy (18), Spain (19), United States (20), and so on. RBS-R was adopted in a study (21) of 163 Chinese children aged 3–8 with ASD enrolled, indicating that a 5-factor model was more appropriate for evaluating RRBs, with a Comparative Fit Index (CFI) of 0.99 and a Goodness of Fit Index (GFI) of 0.73. Another reliability and validity study (16) of RBS-R from Canada showed that the goodness-of-fit indices of the hypothesized latent-factor models of the RBS-R failed to achieve anticipated success. Another study in the United States found that the four- and six-factor models both demonstrated adequate-to-good fit in a sample of 350 children with ASD aged 2 to 9 years (20). In conclusion, the psychometric analysis of the RBS-R was confirmed with different nations and samples.

Since the RBS-R is a widely used analytical tool for researching the RRBs of ASD, it is crucial to clarify the appropriateness of using the RBS-R in Chinese children with ASD. Currently, there are only two domestic studies on the reliability and validity of RBS-R in ASD. In our study, we aimed to increase the sample to measure the reliability and validity of the RBS-R among children with ASD aged 2–4 years in Jiangxi province of China. According to Rouquette A (22), a minimum sample size of 300 subjects is generally acceptable but should be increased when the number of factors is large. This research was also designed to determine the feasibility of employing the RBS-R as an assessment tool for the identification and diagnosis of ASD in the future.

## Materials and methods

### Participants and procedures

We recruited children aged 2–4 years old from a hospital in Jiangxi province. Inclusion criteria were: (1) native Jiangxi province children, (2) no parent-reported visual and auditory impairment, epilepsy, cerebral palsy, trisomy 21 syndrome, or other psychiatric diseases. These children were selected from those who received the assessment of the Childhood Autism Rating Scale (CARS) and based on a score of no less than 30. Children were then diagnosed with ASD according to the DSM-5. The parents or caregivers of eligible children were given written informed consent and the demographic questionnaire, RBS-R, and Autism Behavior Checklist (ABC). The researchers verified their eligibility once they completed these questionnaires, and a second RBS-R was issued to the parents who consented to complete the questionnaire a second time one week after the first one was completed. When conducting the assessment, researchers would prompt participants to complete any incomplete questionnaires.

### Measures instrument

The original Repetitive Behavior Scale (RBS) had three subscales: (a) Stereotypic Behavior, (b) Self-injurious Behavior, and (c) Compulsions which was established by Bodfish (23). RBS-R-based RBS was built by Bodfish in 2020 (15), which was intended to assess the variety of RRBs observed in individuals with ASD and the 43 items can be categorized into the six following subscales: stereotyped behavior (6 items), self-injurious behavior (8 items), compulsive behavior (8 items), ritualistic behavior (6 items), sameness behavior (11 items), and restricted behavior (4 items). All items are rated on a four-point Likert scale of severity; the higher the scores, the more severe the conditions (i.e., 0 = behavior does not occur, 1 = behavior occurs and is a mild problem, 2 = behavior occurs and is a moderate problem, 3 = behavior occurs and is a severe problem). The Chinese version was cited by Li et al. (24) and translated from English to Simplified Chinese by two native Chinese speakers who majored in developmental-behavioral pediatrics and were also fluent in English. To guarantee the consistency of the message, two professors then proofread and translated the Chinese version back into English. In our study, we added descriptions of some items by utilizing more pertinent and detailed explanations from knowledgeable doctors who evaluated numerous individuals with ASD. The RBS-R used in this study was completed by the parents/caregivers of the participants during the evaluation of CARS. The questions on this scale were given to the parents/caregivers to respond to with reference to the preceding month.

### Data analysis

Confirmatory factor analysis (CFA) was applied to test the overall fit of the data to the scale model with 43 items. Statistical indices used to evaluate the model fits include the model of chi-square/degrees of freedom (χ2/df), the comparative fit index (CFI), the goodness of fit index (GFI), the root mean square error of approximation (RMSEA), the standardized root means square residual (SRMR), and the Akaike information criterion (AIC). The index criteria for well-fitting models were: CFI > 0.9, GFI > 0.9, RMSEA < 0.08, and 2 < χ2/df < 5 (25). The reliability of the RBS-R was determined by the test-retest reliability and internal consistency. Test-retest reliability was estimated by an intra-class coefficient (ICC), which was calculated by the correlation between the first and second completion of the scale. The recommended ICC value is ≥0.7, and a value of ≥0.6 is considered acceptable (26). Cronbach’s alpha was used to determine the internal consistency of the scale. An acceptable cutoff value is not below 0.7 (27). The CFA was performed using AMOS, version 17, and SPSS, version 19 was used for other statistical analyses.

## Result

### Demographic characteristics

The sample of the current study consisted of 381 Chinese children with ASD, including 305 boys and 76 girls, with a mean age of 3.14 years old (range 2–4.92). The mean CARS score was 36.04 (range 30–44). The descriptive statistics for these subjects, including both demographic and diagnostic information, are shown in Table 1.

**TABLE 1:** Socio-demographic and diagnostic characteristics.

Demographic and diagnostic variables	*N*	Percentage (%)
**Age**		
2–3	189.00	49.61
3–4	119.00	31.23
4–5	73.00	19.16
**Gender**		
Boy	305.00	80.05
Girl	76.00	19.95
**The severity of autism spectrum disorder**		
Mild	144.00	37.80
Severe	225.00	59.06
Missing	12.00	3.15
**Age of father**		
<30	146.00	38.32
30–40	165.00	43.31
≥40	44.00	11.55
Missing	26.00	6.82
**Age of mother**		
<30	206.00	54.07
30–40	134.00	35.17
≥40	17.00	4.46
Missing	24.00	6.30
**Education level of father**		
Junior high school diploma or below	116.00	30.45
High school diploma	160.00	41.99
Undergraduate degree	72.00	18.90
Graduate degree or above	16.00	4.20
Missing	17.00	4.46
**Education level of mother**		
Junior high school diploma or below	137.00	35.96
High school diploma	137.00	35.96
Undergraduate degree	78.00	20.47
Graduate degree or above	12.00	3.15
Missing	17.00	4.46

The severity of autism spectrum disorder was evaluated by the score of CARS. 30–35 was defined as mild and ≥36 was defined as severity.

### Item analysis

Table 2 provides details on the positive response indices, frequency of endorsement, and spearman’s correlation coefficients between the components of the total score. The frequency of positive responses ranged between 1.31% (i.e., for item 13: Inserts finger/object) and 77.17% (i.e., for item 4: locomotion). The correlation between items of total score ranged between 0.123 and 0.487.

**TABLE 2:** Frequency of endorsement (affirmative answer) for items of the RBS-R and the spearman’s correlation coefficients between items of the total score.

Item	Frequency of endorsement in subscale (%)		Mean	SD	*r*
Stereotypy subscale		96.85			0.633**
1	Body movements	12.60	0.17	0.48	0.262**
2	Head movements	21.52	0.26	0.53	0.326**
3	Finger movements	36.22	0.57	0.90	0.395**
4	Locomotion	77.17	1.38	1.04	0.361**
5	Object usage	81.10	1.48	0.98	0.308**
6	Sensory	61.15	0.94	0.92	0.441**
Self-injurious subscale		33.86			0.394**
7	Hits w/body	18.37	0.25	0.60	0.290**
8	Hits against surface	13.91	0.18	0.51	0.200**
9	Hits w/object	3.15	0.03	0.17	0.274**
10	Bites self	6.04	0.08	0.37	0.176**
11	Pulls hair/skin	2.89	0.04	0.24	0.199**
12	Rubs/scratches	1.84	0.02	0.16	0.123**
13	Inserts finger/object	1.31	0.02	0.18	0.175**
14	Picks skin	3.67	0.06	0.31	0.236**
Compulsive subscale		68.50			0.713**
15	Ordering	28.08	0.42	0.76	0.442**
16	Completeness	19.69	0.30	0.69	0.461**
17	Washing	6.56	0.09	0.35	0.236**
18	Checking	4.99	0.07	0.33	0.327**
19	Counting	5.25	0.07	0.31	0.353**
20	Hoarding	5.77	0.07	0.30	0.374**
21	Repeating	37.01	0.53	0.78	0.271**
22	Needs to touch/tap	21.26	0.28	0.60	0.329**
Ritualistic subscale		82.94			0.617**
23	Eating/mealtime	34.38	0.54	0.86	0.334**
24	Sleeping/bedtime	24.93	0.37	0.71	0.328**
25	Self-care routine	6.56	0.09	0.35	0.365**
26	Transportation routine	12.86	0.18	0.51	0.447**
27	Play/leisure routine	17.06	0.20	0.48	0.478**
28	Communication	65.62	1.07	0.98	0.175**
Sameness subscale		71.92			0.761**
29	Placement of objects	8.92	0.11	0.39	0.331**
30	No new places	14.17	0.17	0.44	0.383**
31	No interruption	38.06	0.49	0.71	0.421**
32	Walks certain way	8.92	0.12	0.41	0.412**
33	Sits certain place	4.99	0.06	0.30	0.256**
34	Appearance/behavior of others	6.82	0.11	0.45	0.257**
35	Uses certain door	2.62	0.03	0.22	0.384**
36	Videotapes	34.12	0.57	0.90	0.487**
37	Difficult transitions	28.61	0.35	0.62	0.467**
38	Insists on routine	10.50	0.13	0.43	0.271**
39	Insists on time	3.67	0.04	0.23	0.324**
Restricted subscale		77.69			0.620**
40	Preoccupation with subject	43.04	0.72	0.96	0.411**
41	Attached to object	30.18	0.50	0.85	0.366**
42	Preoccupied with part of object	38.58	0.64	0.93	0.283**
43	Preoccupation with movement	43.57	0.70	0.94	0.413**

**P ≤ 0.01.

### Confirmatory factor analysis

**FIGURE 1:**
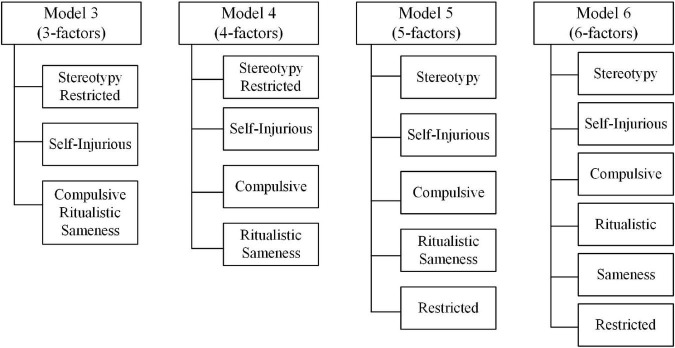
The 3–6 factors competing hypothesized models of the RBS-R factor structure.

We hypothesized a range of three to six factors for the specific models, as shown in Figure 1.

The CFA results for all four models (i.e., Models 3, 4, 5, and 6) in Table 3, indicated that the 3-, 4-, 5-, and 6-factor models were all reasonably good fits for the data, based on the fit statistics guidelines previously described. However, considering the optimal statistical fit, we suggested the 6-factor to be the best model. The standardized factor loadings of CFA, ranging from 0.094 to 0.658, are shown in Table 4.

**TABLE 3:** Goodness-of-fit indices for the hypothesized latent factor RBS-R models (*N* = 381).

	χ^2^	df	χ^2^/df	CFI	GFI	RMSEA	SRMR	AIC	BIC
Model 3	1539.768	849.000	**1.814**	0.703	0.840	**0.046**	**0.062**	1733.768	2116.474
Model 4	1458.895	848.000	**1.720**	0.737	0.847	**0.043**	**0.060**	1654.895	2041.546
Model 5	1437.682	845.000	**1.701**	0.745	0.849	**0.043**	**0.059**	1639.682	2038.169
Model 6	1411.453	840.000	**1.680**	0.754	0.852	**0.042**	**0.054**	1623.453	2041.667

RBS-R, behavior scale-revised; CFI, comparative fit index; GFI, the goodness of fit index; RMSEA, Root-mean-square error of approximation; SRMR, standardized root-mean-square residual; AIC, Akaike information criterion; BIC, Bayes information criterion; The numbers in bold meet the criterion set for good model fit.

**TABLE 4:** The standardized factor loadings of the confirmatory factor for RBS-R.

	Item	Factor-1	Factor-2	Factor-3	Factor-4	Factor-5	Factor-6
1	Body movements	0.346					
2	Head movements	0.414					
3	Finger movements	0.554					
4	Locomotion	0.437					
5	Object usage	0.201					
6	Sensory	0.501					
7	Hits w/body		0.429				
8	Hits against surface		0.461				
9	Hits w/object		0.470				
10	Bites self		0.552				
11	Pulls hair/skin		0.449				
12	Rubs/scratches		0.158				
13	Inserts finger/object		0.521				
14	Picks skin		0.374				
15	Ordering			0.409			
16	Completeness			0.401			
17	Washing			0.324			
18	Checking			0.412			
19	Counting			0.497			
20	Hoarding			0.502			
21	Repeating			0.172			
22	Needs to touch/tap			0.261			
23	Eating/mealtime				0.251		
24	Sleeping/bedtime				0.331		
25	Selfcare routine				0.347		
26	Transportation routine				0.619		
27	Play/leisure routine				0.658		
28	Communication				0.094		
29	Placement of objects					0.338	
30	No new places					0.365	
31	No interruption					0.396	
32	Walks certain way					0.502	
33	Sits certain place					0.368	
34	Appearance/behavior of others					0.284	
35	Uses certain door					0.575	
36	Videotapes					0.381	
37	Difficult transitions					0.515	
38	Insists on routine					0.275	
39	Insists on time					0.446	
40	Preoccupation with subject						0.506
41	Attached to object						0.397
42	Preoccupied with part of object						0.221
43	Preoccupation with movement						0.362

### Reliability statistics

Table 5 depicts the internal consistency (Cronbach’s a) indices and test-retest correlations for the RBS-R and each factor. The internal consistency for the total score on the RBS-R was 0.834, and the internal consistency for the factors ranged from 0.744 to 0.83. The aforementioned findings showed that this scale has strong internal consistency. A retest was conducted on 24 participants who had previously taken the RBS-R in order to evaluate the instrument’s consistency and reliability The test-retest reliability was good to excellent for the total score of the RBS-R (0.749) and each factor (factor 1: 737, factor 2:0.829, factor 3:0.639, factor 4:0.754, factor 5:0.607, and factor 6:0.689). As for the reliability of the RBS-R, the results indicated a high internal consistency of all subscales and the total score of the RBS-R in this study.

**TABLE 5:** The internal consistency and test-retest reliability of the RBS-R.

	Internal consistency	Test-retest
Factor1	0.830	0.737
Factor2	0.765	0.829
Factor3	0.744	0.639
Factor4	0.783	0.754
Factor5	0.751	0.607
Factor6	0.811	0.689
Total	0.834	0.749

## Discussion

This study aimed to evaluate the psychometric properties of the RBS-R in patients with ASD in Jiangxi Province. If the RBS-R has strong validity and reliability, it will be used to gauge the severity of RRBs in patients with ASD and our findings confirmed the utility of the RBS-R as a measure of a wide range of RRBs seen in 2–4 years old children with ASD from Jiangxi province of China. This study supports Li’s (21) assertion that the original RBS-R items may be modified to fit the Chinese context.

The Self-Injurious behaviors (33.86%) exhibited the lowest frequency behaviors compared to other subscales in our study, which was consistent with most studies from other countries such as China (21) and America (20). However, the frequencies were much lower for each item of this subscale compared to the results of He et al. (21) with 12 (rubs/scratches) of 1.84% in our study and 13.5% in other Chinese participants (21), 18.5% in Spanish participants (19), 16.3% in United States participants with ASD (20), and 2.7% in Italian participants with ASD (18). These behaviors of item 9 (hits w/object), item 11 (Pulls hair/skin), item 12 (rubs/scratches), and item 13 (inserts finger/object) were infrequent during the assessment of CARS according to the reports from parents or caregivers. The highest frequency of item was item 4 (locomotion), which was 77.17% in the study. Among Spanish-speaking patients with ASD, the highest frequency was 50.2% from item 28 (communication) (19). The frequency of item 28 (communication) in our report was 65.62%, higher than the former. In an American study, the highest frequency was 67.7%, from item 40 (preoccupation with one subject) (20), suggesting that different samples and cultures can explain the differences in frequencies.

We proposed the structural models with three to six factors according to some literature (16). In accordance with the statistical indices’ guidelines for the structural equation model, these models were all reasonably good fits for our sample. The best model that fit well was the 6-factors model with the ratio between χ2 and the degrees of freedom (χ2/df) of 1.68, the CFI of 0.754, the GFI of 0.852, and the RMSEA of 0.042. The standardized factor loading ranged from 0.094 to 0.658, some items of which were lower than the cutoff. According to Kline’s criterion, a factor loading cutoff of ≥0.35 was applied (28). However, the index did not become much better after removing these three items, therefore, we reserved item 28 (communication), item 12 (rubs/scratches), and item 21 (repeating), which would be found in ASD children. Additionally, several of the items on this scale overlapped with one another. For example, parents were puzzled by item 31 (no interruption) and item 37 (difficult transitions) as item 31 came along with item 37. Coincidentally, scholars (21) found there were duplicates among the content of certain RBS-R items among the ritualistic subscale and sameness subscale.

The structure validity and internal validity of this scale were accessed using a confirmatory factor in accordance with the theoretical framework proposed by Bodfish (15) and other works of research. Concerning the reliability, the internal consistency for the subscales of the original structure ranged from 0.744 (Compulsive Behavior) to 0.83 (Stereotypic Behavior); the internal consistency for all RBS-R items was 0.834. Test-retest of the RBS-R ranged from 0.607 (Self-injurious subscale) to 0.829 (Self-injurious subscale); the test-retest for all RBS-R items was 0.749. These results indicated an acceptable internal consistency of all subscales and the total score of the RBS-R in this study. The internal consistency estimated by Cronbach’s alpha value was lower than a study by He et al. (21) and higher than another study by Li et al. (24). The diverse samples used in the experiments may be the cause of the inconsistent outcomes. But according to these three investigations, the RBS-overall R’s score and all of its subscales demonstrated acceptable levels of internal consistency.

### Strengths and limitations

The first of the two strengths of our study is that we recruited 381 ASD children from 2 to 4 years old to ensure the sample size was sufficient to support the use of CFA, which was more than fivefold the number of items. Second, we collected these data of RBS-R while the assessment of CARS, so that evaluators were able to assess RRBs of ASD children face-to-face and we would check the scale to ensure that caregivers had finished the questionnaire, leading to reduced reporting errors from caregivers. The first limitation of our study was that we only obtained children 2–4 years old, in which, the behaviors from the compulsive subscale and sameness subscale may not function effectively. Secondly, we had not determined how this scale’s psychometric characteristics varied according to factors like age, gender, cognitive capacity, and so on, since is known to be age-related and dependent on the intellectual functioning of individuals with ASD (29).

## Conclusion

In conclusion, using the original framework of RBS-R to assess the severity of repetitive and stereotypic behaviors in young Chinese children with ASD from Jiangxi Province would enable a better differential diagnosis. Future studies should expand the sample, focusing in particular on Chinese school-aged children with ASD.

## Data availability statement

The raw data supporting the conclusions of this article will be made available by the authors, without undue reservation.

## Ethics statement

The studies involving human participants were reviewed and approved by the Ethics Committee of Jiangxi Provincial Children’s Hospital. Written informed consent to participate in this study was provided by the participants’ legal guardian/next of kin.

## Author contributions

XL, XZ, and SZ designed the study. XL, YX, MG, LH, and ZL performed the study. XL wrote and reviewed the manuscript. All authors contributed to the article and approved the submitted version.
