# Supplementary material for: Reliability and validity of the repetitive behavior scale-revised for young Chinese children with autism spectrum disorder in Jiangxi Province
Source: Front Pediatr. 2022 Sep 8;10:939841. doi: 10.3389/fped.2022.939841 (PMC9492994; doi:10.3389/fped.2022.939841)
Supplement: Supplementary file 1 [file Data_Sheet_1.PDF]

Table 1 correlation analysis between items

Item	RBSR1	RBSR2	RBSR3	RBSR4	RBSR5	RBSR6	RBSR7	RBSR8	RBSR9	RBSR10	RBSR11
RBSR1	1.000										
RBSR2	0.333**	1.000									
RBSR3	0.197**	0.203**	1.000								
RBSR4	0.091*	0.107*	0.202**	1.000							
RBSR5	0.094*	0.004	0.081	0.189**	1.000						
RBSR6	0.108*	0.135**	0.302**	0.194**	0.067	1.000					
RBSR7	0.051	0.149**	0.175**	0.077	0.100*	0.117*	1.000				
RBSR8	0.052	0.149**	0.164**	0.050	0.056	0.054	0.202**	1.000			
RBSR9	0.068	0.183**	0.132**	0.086	0.034	0.065	0.224**	0.181**	1.000		
RBSR10	0.043	0.062	0.031	0.096*	-0.032	0.060	0.119*	0.164**	0.209**	1.000	
RBSR11	0.123*	0.128*	0.061	0.055	-0.052	0.045	0.122*	0.244**	0.325**	0.286**	1.000
RBSR12	0.005	-0.026	0.050	-0.030	0.017	0.054	0.046	-0.001	0.198**	0.130*	0.207**
RBSR13	0.089	0.114*	0.073	0.063	-0.049	0.040	0.244**	0.164**	0.374**	0.168**	0.253**
RBSR14	0.182**	0.138**	0.138**	0.085	-0.002	0.103*	0.116*	0.159**	0.205**	0.125*	0.135**
RBSR15	0.121*	0.184**	0.062	0.038	0.102*	0.100*	0.082	0.082	0.046	-0.019	0.092
RBSR16	0.120*	0.121*	0.133**	0.053	0.089	0.207**	0.111*	0.024	0.131**	-0.044	-0.013
RBSR17	0.052	0.011	0.120*	-0.040	-0.107*	0.091	0.062	0.016	0.137**	0.018	0.077
RBSR18	0.018	0.002	0.094	-0.033	-0.001	0.060	0.105*	-0.024	0.098	-0.009	0.031
RBSR19	0.116*	0.097	0.074	0.012	0.003	0.059	0.037	0.072	0.160**	-0.011	0.101*
RBSR20	0.068	0.084	0.055	0.004	-0.027	0.077	0.109*	-0.037	0.214**	0.080	0.093
RBSR21	0.016	0.011	-0.030	0.083	0.219**	0.005	-0.011	0.021	0.044	0.060	0.016
RBSR22	0.198**	0.162**	0.111*	0.027	0.017	0.219**	0.113*	-0.046	0.126*	0.094	0.060
RBSR23	-0.019	0.082	0.084	0.027	0.049	0.200**	0.105*	0.048	-0.020	-0.017	-0.038
RBSR24	0.045	0.026	0.068	-0.038	0.035	0.135**	0.117*	0.037	0.080	0.052	0.038
RBSR25	0.055	0.145**	0.116*	0.084	0.050	0.140**	0.137**	0.011	0.129*	0.106*	0.078
RBSR26	0.018	0.071	0.070	-0.003	0.035	0.150**	0.062	0.019	0.153**	0.067	0.071
RBSR27	0.158**	0.104*	0.099*	0.035	0.019	0.175**	0.122*	0.016	0.071	-0.004	0.089
RBSR28	-0.019	0.019	0.063	-0.016	-0.038	0.031	-0.081	-0.089	-0.006	-0.054	-0.056
RBSR29	0.070	0.063	0.031	0.059	0.032	0.042	0.038	0.002	0.097	-0.041	0.000
RBSR30	0.180**	0.154**	0.141**	0.044	-0.005	0.115*	0.108*	0.013	0.101*	0.053	0.061
RBSR31	0.099*	0.087	0.070	0.044	0.175**	0.036	0.042	0.056	0.096	0.010	0.070
RBSR32	0.043	0.095	-0.047	0.170**	0.037	0.052	0.125*	0.006	0.101*	0.036	0.159**
RBSR33	-0.085	-0.001	-0.037	0.013	-0.026	0.081	0.047	-0.025	0.095	0.040	0.031
RBSR34	-0.040	0.005	0.024	0.091	0.076	0.062	0.083	0.062	0.242**	0.142**	0.013
RBSR35	0.032	0.066	0.044	0.067	0.079	0.104*	0.094	0.069	0.159**	0.025	0.167**
RBSR36	0.070	0.080	0.116*	0.069	0.046	0.187**	0.111*	0.060	0.064	0.101*	0.136**
RBSR37	0.075	0.112*	0.081	0.051	0.076	0.099*	0.041	-0.007	0.019	-0.003	0.052
RBSR38	-0.029	0.111*	0.048	0.006	-0.079	0.069	0.032	0.006	0.181**	0.058	0.040
RBSR39	-0.073	0.028	0.089	-0.015	0.007	0.104*	0.094	0.038	0.203**	0.065	0.213**
RBSR40	0.192**	0.052	0.034	-0.017	0.017	0.153**	0.114*	-0.011	0.069	0.050	.118*
RBSR41	0.096*	0.129**	0.073	0.076	0.001	0.125**	0.036	-0.001	0.013	0.003	0.037
RBSR42	0.101*	0.093	0.043	0.081	0.080	0.055	0.082	0.052	0.047	-0.052	0.009
RBSR43	0.110*	0.115*	0.132**	0.109*	0.070	0.157**	0.095*	0.051	0.093	0.023	0.072

**P＜0.001; *P＜0.05

Table 1 (Continued)

Item	RBSR12	RBSR13	RBSR14	RBSR15	RBSR16	RBSR17	RBSR18	RBSR19	RBSR20	RBSR21	[illegible]
RBSR1											
RBSR2											
RBSR3											
RBSR4											
RBSR5											
RBSR6											
RBSR7											
RBSR8											
RBSR9											
RBSR10											
RBSR11											
RBSR12	1.000										
RBSR13	0.155**	1.000									
RBSR14	0.075	0.221**	1.000								
RBSR15	0.087	0.117*	0.076	1.000							
RBSR16	0.022	0.150**	0.108*	0.207**	1.000						
RBSR17	0.122*	0.150**	0.062	0.110*	0.106*	1.000					
RBSR18	0.144**	0.077	0.017	0.064	0.210**	0.135**	1.000				
RBSR19	0.054	0.181**	.138**	0.187**	0.148**	0.318**	0.164**	1.000			
RBSR20	0.131*	0.166**	0.068	0.115*	0.187**	0.118*	0.358**	0.194**	1.000		
RBSR21	0.076	-0.045	-0.026	0.029	0.091	-0.031	0.114*	0.006	0.102*	1.000	
RBSR22	0.071	-0.007	0.119*	0.090	0.160**	0.073	0.188**	0.072	0.239**	0.098*	
RBSR23	0.023	0.077	-0.004	0.134**	0.136**	0.152**	0.035	0.088	0.086	-0.011	
RBSR24	0.129**	0.028	0.048	0.140**	0.131**	0.171**	0.049	0.096	0.085	0.030	
RBSR25	0.040	0.150**	0.115*	0.212**	0.238**	0.136**	0.084	0.261**	0.243**	0.109*	
RBSR26	0.228**	0.021	0.053	0.155**	0.046	0.120*	0.193**	0.297**	0.295**	0.076	
RBSR27	0.185**	0.123*	0.123*	0.266**	0.164**	0.231**	0.208**	0.380**	0.240**	0.016	
RBSR28	-0.002	-0.026	0.023	0.076	0.119**	-0.018	0.060	0.107*	0.077	0.031	
RBSR29	0.156**	0.042	0.085	0.346**	0.268**	0.211**	0.136**	0.251**	0.156**	0.029	
RBSR30	0.054	0.155**	0.121*	0.143**	0.164**	0.042	0.183**	0.140**	0.253**	0.076	
RBSR31	0.111*	0.035	0.049	0.258**	0.158**	0.049	0.083	0.113*	0.118*	0.189**	
RBSR32	0.023	0.120*	0.035	0.148**	0.181**	0.098	0.103*	0.215**	0.201**	0.111*	
RBSR33	0.057	0.078	0.079	0.199**	0.103*	0.086	0.112*	0.316**	0.148**	0.034	
RBSR34	0.037	0.142**	0.056	0.099*	0.136**	0.101*	0.081	0.123*	0.108*	0.056	
RBSR35	0.098	0.123*	0.137**	0.184**	0.096	0.091	0.116*	0.328**	0.311**	0.084	
RBSR36	0.086	0.055	0.093	0.187**	0.222**	0.127**	0.079	0.207**	0.106*	0.070	
RBSR37	0.034	-0.024	0.027	0.221**	0.086	0.065	0.173**	0.257**	0.177**	0.156**	
RBSR38	0.076	0.186**	0.154**	0.113*	0.135**	0.145**	0.154**	0.215**	0.208**	0.110*	
RBSR39	0.179**	0.099	0.106*	0.180**	0.084	0.173**	0.214**	0.265**	0.252**	0.120*	
RBSR40	0.072	0.038	0.033	0.144**	0.105*	0.063	0.083	0.115*	0.106*	0.050	
RBSR41	0.010	0.028	0.142**	0.174**	0.153**	.098*	0.059	0.113*	0.181**	-0.037	
RBSR42	-0.070	0.035	0.109*	0.100*	0.153**	-0.019	0.007	0.039	0.084	0.112*	
RBSR43	0.127**	0.074	0.040	0.040	0.156**	0.032	0.160**	0.057	0.107*	0.202**	

**P＜0.001; *P＜0.05

Table 1 (Continued)

Item	RBSR23	RBSR24	RBSR25	RBSR26	RBSR27	RBSR28	RBSR29	RBSR30	RBSR31	RBSR32
RBSR1										
RBSR2										
RBSR3										
RBSR4										
RBSR5										
RBSR6										
RBSR7										
RBSR8										
RBSR9										
RBSR10										
RBSR11										
RBSR12										
RBSR13										
RBSR14										
RBSR15										
RBSR16										
RBSR17										
RBSR18										
RBSR19										
RBSR20										
RBSR21										
RBSR22										
RBSR23	1.000									
RBSR24	0.164**	1.000								
RBSR25	0.115*	0.130**	1.000							
RBSR26	0.086	0.146**	0.214**	1.000						
RBSR27	0.161**	0.264**	0.290**	0.451**	1.000					
RBSR28	0.041	0.042	0.004	0.045	0.045	1.000				
RBSR29	0.088	0.133**	0.134**	0.167**	0.274**	0.121*	1.000			
RBSR30	0.098*	0.147**	0.220**	0.134**	0.254**	0.099*	0.162**	1.000		
RBSR31	0.069	0.131**	0.094	0.093	0.195**	0.022	0.132**	0.151**	1.000	
RBSR32	0.176**	0.053	0.138**	0.134**	0.199**	0.020	0.159**	0.084	0.145**	1.000
RBSR33	0.137**	0.110*	0.275**	0.382**	0.244**	0.012	0.212**	0.078	0.091	0.188**
RBSR34	0.021	0.135**	0.220**	0.261**	0.174**	-0.015	0.126*	0.065	0.023	0.097
RBSR35	0.069	0.148**	0.214**	0.277**	0.238**	0.007	0.285**	0.260**	0.147**	0.304**
RBSR36	0.175**	0.143**	0.217**	0.180**	0.184**	0.022	0.166**	0.098*	0.116*	0.131**
RBSR37	0.136**	0.127**	0.213**	0.173**	0.338**	-0.015	0.191**	0.232**	0.446**	0.206**
RBSR38	0.073	0.073	0.431**	0.214**	0.217**	0.047	0.123*	0.284**	0.085	0.101*
RBSR39	0.114*	0.183**	0.166**	0.268**	0.276**	0.076	0.081	0.159**	0.179**	0.187**
RBSR40	0.135**	0.145**	0.113*	0.247**	0.275**	-0.022	0.162**	0.122*	0.086	0.077
RBSR41	0.066	0.096*	0.128**	0.162**	0.210**	0.073	0.132**	0.196**	0.126**	0.172**
RBSR42	-0.005	-0.001	0.058	0.089	0.084	0.039	0.091	0.107*	0.055	0.072
RBSR43	0.036	0.026	0.117*	0.166**	0.102*	-0.006	0.018	0.124**	0.059	0.178**

**P＜0.001; *P＜0.05

Table 1 (Continued)

Item	RBSR34	RBSR35	RBSR36	RBSR37	RBSR38	RBSR39	RBSR40	RBSR41	RBSR42	RBSR43
RBSR1										
RBSR2										
RBSR3										
RBSR4										
RBSR5										
RBSR6										
RBSR7										
RBSR8										
RBSR9										
RBSR10										
RBSR11										
RBSR12										
RBSR13										
RBSR14										
RBSR15										
RBSR16										
RBSR17										
RBSR18										
RBSR19										
RBSR20										
RBSR21										
RBSR22										
RBSR23										
RBSR24										
RBSR25										
RBSR26										
RBSR27										
RBSR28										
RBSR29										
RBSR30										
RBSR31										
RBSR32										
RBSR33										
RBSR34	1.000									
RBSR35	0.275**	1.000								
RBSR36	0.109*	0.197**	1.000							
RBSR37	0.088	0.204**	0.138**	1.000						
RBSR38	0.141**	0.151**	0.120*	0.188**	1.000					
RBSR39	0.113*	0.315**	0.072	0.260**	0.152**	1.000				
RBSR40	-0.003	0.033	0.255**	0.125**	0.092	0.019	1.000			
RBSR41	0.012	0.099*	0.158**	0.100*	0.123*	0.018	0.240**	1.000		
RBSR42	-0.005	0.081	0.046	0.095*	0.117*	0.050	0.114*	0.069	1.000	
RBSR43	0.038	0.046	0.162**	0.086	0.048	0.060	0.144**	0.119*	0.179**	1.000

**P＜0.001; *P＜0.05
